# Porcelain Inlays

**Published:** 1897-06

**Authors:** N. S. Jenkins

**Affiliations:** Dresden


### Porcelain Inlays.

BY N. S. JENKINS, D.D.S., DRESDEN.

Ever since the treatment of diseased teeth has been regarded as a science, dentists have been seeking for a material for filling cavities which should, in color resemble, and in durability surpass the teeth themselves. Great hopes were entertained some forty years ago, that gutta percha could be so combined with metalic oxides, or other substances, as to provide such a filling, so that, at a time when the rubber dam was unknown, much ingenuity was expended upon this material, and, in the hands of capable men, excellent results were now and then obtained. But this filling, useful as it was and is, in its proper place, soon came to be discarded for permanent use, and the discovery of cohesive gold, together with the application of the rubber dam, turned the thoughts of the profession in the direction of using this noble material for the restoration of decayed and broken down teeth, with results which were indeed marvellous. For many years a good dentist was judged by his ability to manipulate gold with dexterity, and it is only in these later and more enlightened times, when dentistry has taken its proud position as a true branch of the broad science of medicine, that there has arisen the distinction in the mind of the profession, and, to some extent in the mind of the public, between the clever operator and the accomplished dental surgeon. The former may exist without being the latter. The latter cannot exist without being the former.

It is needless to enlarge upon the hopes excited by the discovery of oxychloride and phosphate of zinc. At that time the leading dentists were engaged in perfecting their ingenious processes for the more perfect working of gold, and these materials fell naturally into the hands of more unskilful men, since tha masters disdained a putty like material which any fool, they thought, could use as well as a wise man. Even when really scientific men began to experiment in the improvement of these materials, it was found that, however carefully manufactured, they could not endure the forces of chemical or mechanical disintegration, if standing unprotected, and that there was no hope whatever of making them translucent, so as properly to represent the color of the natural teeth.

But when, at last, porcelain and glass fillings were introduced, it seemed as if the disideratum was approximately reached. Here were materials which represented the color of the natural teeth with reasonable fidelity, and which were good non-conductors. Porcelain could be ground to fit a cavity, or melted in a platinum impression. Many good results were obtained with both these materials, but, in general, they were found to be very inconstant. It tried the skill and patience of the ablest and most enduring men to grind porcelain inlays to a perfect fit, and when either porcelain or glass was melted, it was liable to be disappointing in density or color, and accurate contouring seemed to be only a happy accident. Moreover, one could not depend upon the endurance of the color, and a black line frequently appeared at the junction of filling and enamel.

"I know not what others think, but as for me" I was fascinated with the possibility of glass fillings. I struggled with that seductive and treacherous substance in season and out of season, until I was often forced to exclaim, in the words of Hanns Breitmann, "der Schnitzerl sei verdammt for teaching me dos dings." But at last I realized that a new material must be evolved, one which combined the good qualities of both porcelain and glass, and to its evolution my laboratory has been devoted for the past three years. The problem I set myself was, to make a low melting porcelain, which should retain its color, could be melted to a perfect fusion in a gold inlay, and which would have a clean edge so hard that it would not fracture in setting, nor in enduring the strain of mastication. This result I have at last attained.

Our most successful experiments in pure glass were made by my partner, Dr. O'Brian. Among other things, he discovered that, after a glass filling had been melted, its surface could be immensely improved by placing upon it a thin plate of clear glass and melting once more. By this means a surface was obtained so smooth, and so nearly representing the enamel, that the most beautiful operations could be made. Only, as in all glass fillings, contours were not certain, overflow easily occurred, and perfect manipulation required much care, experience and skill. Besides which, the porosity of the underlying glass made permanency of color uncertain. This method is, however, an extremely valuable one. Every now and then a case occurs where the conditions require just such a surface as can be obtained only in this manner.

In making an inlay filling, the first step is to properly prepare the cavity. There must be no undercuts, or at least only in one direction, and the edges must be clearly defined. A perfect impression must be obtained, employing gold foil for the purpose. Platinum, however thin, and however carefully used, is too intractable for accurate work. For my method, gold foil No. 30 is, in almost all instances, sufficiently thick. For very large and complicated cavities, No. 40 may be taken. The piece of foil must be generously large, and carried, with cotton or spunk, first to the deepest part of the cavity, leaving the edges free; for, of course, the foil will not stretch, but tear, if it is confined to any exterior point as it is being pressed home. Then another and some what larger piece is packed into the cavity, and when this is quite full, then the edges are made, and pressed or burnished until they are perfectly smooth. If there is too much overlapping margin, it can be trimmed away with a sharp lancet. The cotton or spunk, must thereupon be removed, when, with a sharp-pointed excavator, inserted at the most favorable place within the cavity, the gold can be loosened, and gradually worked out in unimpaired condition. The impression thus made is laid upon a creamy bed of powdered asbestos and water, at the bottom of a small, long-handled, platinum or nickel tray, which is gently tapped until the gold has sufficiently imbedded itself in the asbestos. The tray is now held over the blow pipe, and the asbestos completely dried, a condition usually indicated by a loosening from the bottom of the tray. The contents of the tray being sufficiently cooled, the powdered porcelain mixed with water—distilled water is preferable—is placed in the impression, care being taken to have it come exactly to the edges, but not to overflow at any point. It is then built up somewhat higher in the middle, or on those parts which it is desired to contour, an excess of material being necessary to compensate for the consolidation attendant upon melting. After some experience it is possible to get, in most instances, the exact fullness required in one melting, but there is no objection to adding more porcelain and remelting, except loss of time. When the inlay has been properly packed, a cover, which has an opening through which the process can be observed, is put upon the tray, and the whole placed in an asbestos lined muffle, open on one side, and with a round hole in the bottom. Through this hole a gentle heat from the blow pipe is turned on, the moisture in the powdered porcelain thus being slowly evaporated, not boiled out. Then the finger is placed upon the spring of the blow pipe, and under the action of the bellows the heat is very gradually increased until the mass is completely melted. The finished inlay is then removed from its asbestos bed, and the corners of the gold foil gently turned backward until it comes away in a complete piece. Any particles of gold which may adhere to the inlay can be easily removed with the point of an excavator. After trying it in the cavity, to make sure that the fit is perfect, the inlay is usually groved on the inner surface, generally with a small diamond disk. Slight undercuts are made in the cavity, everything is perfectly dried, and the inlay is set with some reliable phosphate of zinc mixed to a thick cream. A little of the phosphate should be smeared within the cavity, as well as upon the bottom of the inlay, the latter being carried at once gently into place. In an approximal cavity I use a piece of tape to bring the filling into position, and at the same time remove the surplus phosphate. In other cavities any convenient method may be employed to remove the surplus, but in all cases it is best to exert the final pressure with a bit of wood, except where it happens as sometimes in a labial cavity, that the thumb nail can be easily used. With firm, but gentle pressure, the inlay is held in place for a few minutes, when it can be left alone, with the rubber dam in position, until the phosphate is completely hardened. In many cases it is safe to cover with varnish, dry with hot air, and remove the dam after a few minutes, but this detail is a matter of judgment. At a subsequent sitting, any slight surplus of overlying phosphate is carefully removed with a fine pointed excavator, when it will be found that the line between the inlay and the edge of the cavity is no wider than such cracks as one often finds in the enamel.

The beauty of this work is only equalled by its practicability. Accurate contours can be made. Of course it is not applicable to all approximal cavities, but in places where its use is indicated, there is no doubt about the condition of the cervical margin, that weak point in obscure cavities, and especially where the pulp is nearly exposed, if intelligently and accurately made, it will stand even better than gold, since it is not supported by, but assists to support thin and frail edges. The operation, although requiring considerable time, is far easier for the patient to bear than the packing of gold. The time of the patient may be saved by taking the impression at one sitting, and making the filling in the patient's absence. To be sure, by carefully following the directions, any ordinary dentist can, in many instances, make in this manner a beautiful and enduring operation, but it is by no means a cheap and easy method. Its proper employment requires the highest qualities of skill, judgement and taste. Only experience will show how the obstacles peculiar to this process are to be overcome, but when this method is once mastered, it gives to the competent dentist a range of operations so wide as to greatly increase his interest in his work, as well as his usefulness to his patients, who immensely appreciate the advantages of this treatment.

Time fails to enlarge upon the use of this porcelain in pivot teeth, crown and bridge work. With it, all the visible part of a broken down molar can usually be restored, with a platinum ring about the neck of the tooth. Malformed roots can be securely crowned, with or without a ring, according to the case, and where the tooth must be thin, because of the closeness of the bite, a far stronger tooth can be made with this material than with a soldered gold backing. When a tooth thus united to a platinum pivot is put under the hammer, it will be found that the tooth will fractue before this porcelain. One great advantage of this new material is found in being able to solder rings, pivots and pins of platinum with gold, and then melt the porcelain without fearing that the gold will melt or flow.

---

Do not look for blemishes continuously, for if you do you'll find them.—*Love's Lance.*

